# The potential of CRISPR/Cas9 genome editing for the study and treatment of intervertebral disc pathologies

**DOI:** 10.1002/jsp2.1003

**Published:** 2018-03-15

**Authors:** Olga Krupkova, Elena Cambria, Lenka Besse, Andrej Besse, Robert Bowles, Karin Wuertz‐Kozak

**Affiliations:** ^1^ Department of Health Sciences and Technology Institute for Biomechanics ETH Zurich Switzerland; ^2^ Department of Oncology and Hematology Cantonal Hospital St Gallen St Gallen Switzerland; ^3^ Department of Bioengineering University of Utah Salt Lake City Utah; ^4^ Department of Orthopaedics University of Utah Salt Lake City Utah; ^5^ Spine Center Schön Klinik München Harlaching Munich Germany; ^6^ Academic Teaching Hospital and Spine Research Institute Paracelsus Private Medical University Salzburg Salzburg Austria; ^7^ Department of Health Sciences University of Potsdam Potsdam Germany

**Keywords:** CRISPR/Cas9, degenerative disc disease, intervertebral disc, low back pain, targeted genome engineering

## Abstract

The CRISPR/Cas9 system has emerged as a powerful tool for mammalian genome engineering. In basic and translational intervertebral disc (IVD) research, this technique has remarkable potential to answer fundamental questions on pathway interactions, to simulate IVD pathologies, and to promote drug development. Furthermore, the precisely targeted CRISPR/Cas9 gene therapy holds promise for the effective and targeted treatment of degenerative disc disease and low back pain. In this perspective, we provide an overview of recent CRISPR/Cas9 advances stemming from/with transferability to IVD research, outline possible treatment approaches for degenerative disc disease, and discuss current limitations that may hinder clinical translation.

## 1 DISC DEGENERATION: THE NEED FOR NOVEL TREATMENTS

Degeneration of the intervertebral disc (IVD) is an age‐related process that is characterized by a catabolic shift, leading to matrix breakdown and—ultimately—structural failure. Apparent degenerative changes first occur in the nucleus pulposus (NP) and are associated with a shift from collagen type II to more fibrotic collagen type I as well as with a reduction in proteoglycans and a consequent loss in hydration and disc height.^1^ However, the annulus fibrosus (AF) also undergoes degenerative changes as evidenced by disorganization of the lamellar structure, possibly leading to structural defects, such as clefts and tears.^2^ The altered biomechanical status during degeneration contributes to the development of tissue damage through the creation of areas of peak stress, exposing the disintegrated tissue to hyperphysiological loading that it cannot withstand.^3^ As the IVD possesses little regenerative capacity, and healing can only take place in the outer AF where nutrient supply is greatest, degeneration gradually progresses without treatment.

Although disc degeneration is a main contributor to back pain, only a subpopulation will become symptomatic and experience so‐called degenerative disc disease (DDD) that is associated with increased expression of inflammatory molecules, including interleukins IL‐1β, IL‐8, and IL‐6 and tumor necrosis factor (TNF)‐α (reviewed in References 4, 5, 6, 7). At the moment, patients suffering from DDD are initially treated conservatively, that is with physiotherapy and analgesic medication, but may have to undergo discectomy if symptoms do not improve. Thus, current therapies only target symptoms but not the underlying molecular processes contributing to disc degeneration and pain development. Accordingly, major effort has been made to design novel, biologically targeted treatment options over the past years, with a focus on 2 approaches:

On the one hand, regenerative therapies to counteract the degeneration process have been attempted but without compelling results thus far. The use of cellular therapies, for example, stem cell treatment, is negatively affected by the harsh microenvironment of the IVD that is characterized by high mechanical loads, inflammatory cytokines, hypoxia, low glucose levels, acidic pH, and high osmolarity.^8^ The application of anabolic substances promoting the production of extracellular matrix (ECM) is hampered by the low cellularity within the IVD (4000 cells/mm^3^ in the NP and 9000 cells/mm^3^ in the AF) and, furthermore, by the fact that these few cells are metabolically not very active.^9^, ^10^ The outcome of injection of classical anabolic factors such as bone morphogenetic protein (BMP)‐7, transforming growth factor (TGF)‐β, or growth differentiation factor (GDF)‐5 is compromised even more by the short half‐life of these growth factors and their rapid diffusion out of the IVD.^8^

On the other hand, numerous recent research activities have focused on the means to modulate inflammation in the IVD, mostly via inhibition of the inflammatory cascade (eg, biologics such as epigallocatechin gallate [EGCG], resveratrol, or piperine)^11^, ^12^, ^13^ or by neutralization of inflammatory mediators (eg, infliximab, a TNF‐α inhibitor^14^). Although these molecular treatments constitute a novel means for molecular disease modulation, their success is likely not sustained, especially as repeated injection of therapeutics into the IVD is not desired.^15^ Novel tools that would allow for safe genetic manipulation of resident cells to modulate the catabolic and inflammatory shift or of therapeutic cells to enhance their robustness, and thus allow them to better withstand the IVD environment, could help to circumvent the limitations of current therapeutic approaches.

## 2 TARGETED GENOME EDITING BY CRISPR/CAS9

Genome editing results in stable phenotype changes and thus can permanently eliminate the underlying causes of diseases. Precise genetic reprogramming techniques have great potential to change traditional “symptomatic” treatments, not only of monogenic diseases but also of age‐related and civilization disorders. In addition, these techniques can be implemented in basic and preclinical research to generate disease phenotypes in vitro and in vivo. To date, known genome editing techniques are based on DNA‐binding nucleases, namely engineered Zinc Finger Nucleases (ZFN) and Transcription Activator‐like Effector Nucleases (TALEN), as well as on RNA‐guided nuclease(s), specifically Clustered Regulatory Interspaced Short Palindromic Repeats‐associated Cas9 (CRISPR/Cas9).^16^ ZFNs and TALENs have been used to directly correct disease‐causing mutations associated with hemophilia B or sickle cell disease. ZFN‐ and TALEN‐based therapies demonstrated success in clinical trials, for example, for HIV, proving the potential of genome editing for applications across basic science, medicine, and biotechnology. However, their main disadvantages are related to the challenges (relatively difficult preparation of functional DNA‐binding nucleases) and costs associated with their design and development.^17^, ^18^ Advantages and disadvantages of ZNF and TALENs are reviewed in Reference 18. Similar to ZFNs and TALENs, CRISPR/Cas9 stimulates a double‐strand DNA break (DSB) at a target genomic locus but is easier to prepare and use and is readily programmable. Thus, CRISPR/Cas9 is regarded as a powerful technique for future mammalian genome engineering.^19^

**Figure 1:**
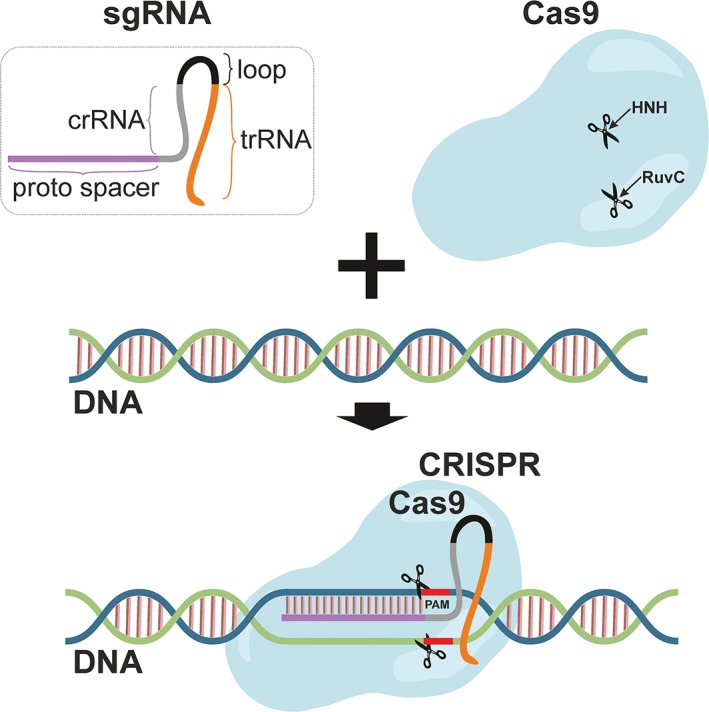
Schematic representation of the CRISPR/Cas9 system. Single‐guide RNA (sgRNA) consists of tracer RNA (trRNA); a loop; crispr RNA (crRNA); and protospacer sequence, which is homologous to the target DNA. wtCas9 possess 2 cleavage activities, HNH and RuvC. CRISPR/Cas9 editing tools consist of sgRNA guiding precisely the Cas9 enzyme to the DNA based on the homology between the protospacer motif and DNA. When the heteroduplex between sgRNA and target DNA is formed, Cas9 performs DNA cleavage in close proximity of the PAM sequence and introduces a double‐strand DNA break

The CRISPR/Cas9 system is composed of the bacterial endonuclease Cas9, which can be directed to any DNA sequence by single‐guide RNA (sgRNA). sgRNA is a short synthetic RNA composed of a scaffold sequence necessary for Cas9‐binding, that is made of crRNA, loop, and trans‐activating crRNA (tracrRNA), and a user‐defined, variable 17‐20 nucleotide spacer that defines the genomic target to be modified.^20^ The short nucleotide spacer of sgRNA is complementary to the genomic DNA target sequence in close proximity to the protospacer adjacent motif (PAM) that, in case of Cas9 obtained from *Streptococcus pyogenes,* is a conserved NGG sequence. However, the PAM is not part of gRNA as it is only present downstream of the target DNA (Figure 1).^21^, ^22^ The recognition of the target DNA is ensured by heteroduplex formation between the nucleotide spacer of sgRNA and the complementary strand of the target DNA, which is followed by Cas9‐mediated DNA cleavage.^23^ Typical wild‐type Cas9 demonstrates double‐stranded DNA cleavage activity provided by 2 domains, RuvC and HNH.^24^ Compared to other components guiding the programmable nuclease to the targeted DNA locus, sgRNA design and synthesis are simple and cost effective. However, a particular concern of CRISPR/Cas9 can be its off‐target activity as the sgRNA can still recognize sequences in the genome with a single‐base mismatch, causing unwanted DSB and mutations. To mitigate this disadvantage, more precise sgRNA designs, synthetically engineered Cas9, or nickase‐Cas9 (Cas9n) with D10A point mutation possessing only single‐stranded DNA cleavage activity have been developed.^25^, ^26^, ^27^ CRISPR/Cas9 has been successfully employed to induce single gene mutations, multiple mutations in one cell,^28^ and to cleave highly methylated regions.^29^ Furthermore, a full range of CRISPR/Cas9 library screening platforms, from genome‐wide to pathway‐specific, is being developed and used to reveal critical biological processes, regulatory genes in development, aging, or drug resistance.^25^, ^30^, ^31^ As such, CRISPR/Cas9 represents a programmable, versatile, and efficient tool for editing virtually any gene. To date, this system has been exploited to reveal exact gene functions, uncover new drug targets, produce more accurate models of human diseases, and provide potential gene correction therapy.^32^, ^33^

**Figure 2:**
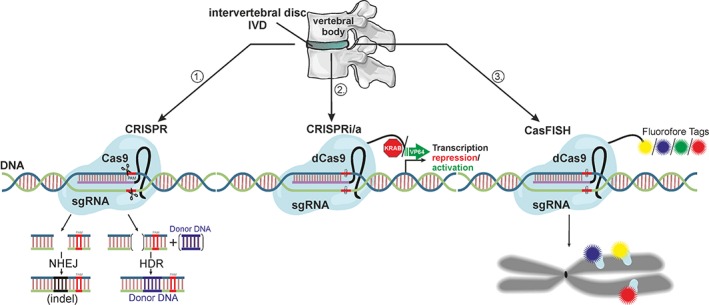
Mechanism of action of CRISPR/Cas9‐based techniques. (1) CRIPSPR/Cas9 gene editing: wtCas9 with both cleavage activities is used to create a double‐strand break on the target DNA, which can be repaired either by nonhomologous end joining (NHEJ) or by homology directed repair (HDR) in case a template DNA is provided. (2) CRISPR/Cas9 interference (i) or activation (a): deathCas9 (dCas9) without cleavage activity is guided to the DNA site around the transcription start site. dCas9 fused with KRAB domain is used for transcription repression, whereas dCas9 fused with VP64 is used for transcription activation of target gene. (3) CasFISH‐mediated chromosome labeling. dCas9 is fused with fluorophore tag and guided in vitro to the target chromosomal DNA that shall be visualized

CRISPR/Cas9‐based techniques can be used not only to disrupt but also to repair and/or regulate gene expression (Figure 2). To generate *CRISPR/Cas9‐mediated knockouts*, RNA‐guided Cas9 induces DSBs, commonly activating the nonhomologous end‐joining (NHEJ) repair pathway. NHEJ produces small random insertions or deletions (indels), resulting in frameshift mutations and loss‐of‐function phenotypes.^34^ *CRISPR/Cas9‐mediated gene editing* is achieved in the presence of template DNA, when DSBs are repaired by so‐called homology‐directed repair (HDR) pathways, which act instead of NHEJ and provide precise insertion of donor DNA into the target site. Apart from site‐specific DNA repair, HDR can aid in generating controlled gene knockouts and inserting marker sequences or resistance genes for further selection of cells with desired phenotypes.^35^ *CRISPR/Cas9‐mediated transcriptional regulation* of gene expression can be achieved by CRISPR interference (CRISPRi) and CRISPR activation (CRISPRa), including CRISPR/Cas9‐mediated epigenetic modification of histones. These techniques utilize catalytically inactive RNA‐guided Cas9 (so‐called dead Cas9, dCas9), fused with transcriptional activators and repressors (VP64 and KRAB, respectively)^36^, ^37^ or with histone‐modifying domains (eg, p300, LSD1) that can regulate transcription by altering chromatin structure.^38^ These gRNA‐dCas9 complexes can be designed to reversibly target specific regulatory sequences, act as a scaffold for various transcriptional factors, or directly interfere with transcription.^17^, ^33^ In addition, CRISPR technology (particularly CRISPR/Cas13) can be applied to edit RNA by targeting Cas13a protein to RNA, instead of DNA.^39^ An overview of possible CRISPR/Cas‐based techniques and their specifications is given in Table 1. CRISPR/Cas9 can be used in basic IVD research to answer fundamental questions on pathway interactions, to simulate IVD pathologies for research and drug development, and possibly to treat DDD.

**Table 1:** Specifications of different CRISPR/Cas‐based techniques

CRISPR/Cas	Cas form	Target sequence	Application	Vectors
CRISPR/Cas9 DNA application				
CRISPR/Cas9 KO	Cas9 or Cas9n	Early exon or evolutionary conserved region	Gene knockout in vitro, in vivo	Viral transduction for stable delivery, RNP complex electroporation for transient delivery
CRISPR/Cas9 GE	Cas9	Cut site ≤30nt from the proximal ends of the repair template	Gene editing in vitro, in vivo	
CRISPRi	dCas9‐KRAB	Targeted to promoters/enhancers	Suppression of gene expression	
CRISPRa	dCAs9‐VP64	Targeted to promoters/enhancers	Activation of gene expression	
CASFISH	dCas9‐fluorophore	Within the desired exon of the gene	In situ labeling of fixed cells in vitro	RNP electroporation
CRISPR/Cas13a RNA application				
CRISPR/Cas13a KO	Cas13a	Early exon or evolutionary conserved region	RNA knockdown	Viral transduction for stable delivery, RNP complex electroporation for transient delivery
CRISPR/Cas13a GE	Cas13a	Around desired adenosine in spliced RNA	ADAR (adenosine to inosin; A‐G) RNA editing	
CRISPR/dCas13a	Cas13a (catalytically inactive)‐fluorophore	Region within specific spliced RNA variant	RNA tracking	

### 2.1 CRISPR/Cas9 in IVD research

#### 2.1.1 Genome targeting

CRISPR/Cas9 is a state‐of‐the‐art tool to determine how the genotype influences the phenotype by revealing details on genetic and epigenetic regulation of cell function. IVD research has been historically hindered by slow proliferation and dedifferentiation of IVD cells as well as by a lack of stabilized cell lines. Recently, immortalized rat NP and AF cell lines have been developed using the rho‐associated kinase (ROCK) inhibitor Y27632.^40^ As kinase inhibitors often have unspecific effects, precise targeting of the ROCK gene by CRISPR/Cas9 could provide an alternative tool for IVD cell immortalization. Another possible application of CRISPR/Cas9 in primary IVD cells can be to prevent their dedifferentiation, a common issue of in vitro studies.^41^ Targeting the expression of dedifferentiation‐associated genes, such as collagen II by CRISPRa or collagen I by CRISPRi, could possibly prolong the use of cultured IVD cells in vitro. Collagen II and aggrecan have recently been targeted by CRISPRa in tissue‐derived stem cells (ASCs) to shift the cell phenotype toward an IVD phenotype and could also be applied directly in IVD cells.^42^

In cartilage research, the Swarm rat chondrosarcoma (RCS) cell line is commonly used to study chondrocyte‐specific phenotype due to its similarity to normal rat cartilage and ability to generate cell clones. Recently, an RCS cell line stably expressing Cas9 (RCS‐Cas9) was developed and used to investigate the function of the ECM molecules aggrecan and hyaluronan.^43^, ^44^ aggrecan knockout in RCS‐Cas9 cells provided additional knowledge on the role of aggrecan in cell attachment, chondrosarcoma formation, and gene regulation,^43^ while knockout of hyaluronan synthase‐2 revealed the essential role of hyaluronan in the assembly of chondrocyte pericellular matrix.^44^ In a chondrogenic mouse teratocarcinoma cell line (ATDC5), CRISPR/Cas9‐mediated deletion of the specific binding site of the microRNA miR‐322, reduced mitogen‐activated protein kinase kinase 1 (MAP2K1 or MEK1) protein levels, and provided new insights into cartilage development.^45^

Similar to these cell lines, IVD cells with stably expressed Cas9 could aid in explaining the relationship between IVD genotype and phenotype in the future. Recent studies on IVD biology highlighted the importance of membrane proteins, namely, of cell surface receptors (eg, toll‐like receptor (TLR) TLR2^46^), channels (eg, aquaporins^47^, transient receptor potential cation channel subfamily V member 4 (TRPV4)^48^), and adhesion molecules (eg, integrins^49^) as these proteins allow IVD cells to sense their environment and respond to its changes. Although the expression and activity of these molecules are altered during DDD, understanding their exact function has been challenging so far as inhibitors/antagonists and activators/agonists are often nonselective or have short half‐lives.^50^, ^51^ The CRISPR/Cas9 system enables the investigation of the exact cell‐ECM interactions by functional knockouts, knockdowns, or knockins of receptors, specific subunits, and interaction partners. As an example, gRNA‐mediated silencing by dCas9‐KRAB‐GFP (CRISPRi) was used to study the role of N‐cadherin (CDH2) in porcine and human NP cells, revealing that CDH2 regulates juvenile NP cell phenotypes during NP cell cluster formation.^52^ Both stable and temporary CRISPR/Cas9 modifications have the potential to uncover the mechanisms involved in ECM turnover (eg, matrix metalloproteinases [MMPs], a disintegrin and metalloproteinase with thrombospondin motifs [ADAMTS]), survival, senescence, and mechanotransduction of IVD cells. In addition, CRISPRi has recently been applied to study the role of cytokine receptors TNFR1 and IL1R1 and their downstream proinflammatory signaling in human primary IVD cells.^53^ Identification and analysis of noncoding regulatory sequences, such as previously identified enhancers of ECM‐degrading enzymes, will also be possible.^54^

Moreover, transduction of target cells with pooled lentiviral libraries carrying multiple gRNA and Cas9 protein or only gRNAs‐Cas9 for defined sets of genes can allow for the functional screening and identification of molecular pathways involved in various diseases,^55^, ^56^, ^57^ possibly including DDD in the future. CRISPR/Cas9 also contributes to the study of genomic regions capable of molecular interactions thanks to the development of CRISPR engineered DNA‐binding molecule‐mediated chromatin immunoprecipitation (enChIP), where a specific antibody is fused to a dCas9 coexpressed with a gRNA.^58^ In IVD research, specifically in studies focusing on IVD oxygenation, enCHiP could, for example, be used to investigate the interaction between hypoxia‐responsive elements (HREs) and hypoxia‐inducible factors such as HIF‐1α.^47^, ^59^ Another use of the CRISPR technology is the visualization of genomic loci in cell nuclei, or so‐called Cas9‐mediated fluorescence in situ hybridization (CASFISH)^60^ (Figure 1). By using multicolor dCas9/sgRNAs, several target loci can be labeled simultaneously,^60^ for example, fibronectin, TGF‐β1, and MMPs in human IVDs.^61^, ^62^ Compared to the traditional method, CASFISH could prevent the disruption of the spatial organization of the genetic elements.^60^

#### 2.1.2 Disease models

CRISPR/Cas9 systems can be used to generate disease models and thus characterize the functionality of identified therapeutic targets and developed drugs. *In vitro disease models* can be prepared from NP and AF cells or organ cultures intended to simulate the degenerative phenotype. It has been demonstrated that in vitro organ cultures can be more suitable than cells to study IVD biology and therapeutic testing as they retain biological and biomechanical properties similar to the IVD in vivo.^63^ However, hallmarks of IVD degeneration are usually not present in organ cultures and have to be induced by nonphysiological insults like injections of proteases^64^ or high concentrations of proinflammatory cytokines.^65^ Generating organ cultures with multiple edited genes (aggrecan, collagen, MMPs, ADAMTS, ILs) might simulate IVD pathologies more naturally. CRISPR/Cas9 constructs have been delivered into tissues through several methods, including viral vectors,^66^ nanoparticles,^67^ or as a gRNA‐Cas9 ribonucleoprotein (RNP) complex by nucleoporation, so this option can be feasible.^68^

Another approach involves deriving tissues from human‐induced pluripotent stem cells (iPSCs) edited with CRISPR/Cas9. This idea has been proposed to develop personalized arthritis therapeutics.^69^ Cartilage can indeed be obtained through chondrogenesis of human iPSCs.^70^ Deficiencies can first be introduced in iPSCs with CRISPR/Cas9, and the engineered cartilage is then subjected to arthritic stimuli such as proinflammatory cytokines or mechanical loading. Candidate drugs can then be screened for their potential to rescue the phenotype. Organ cultures with custom modifications in multiple genes and iPSC‐derived engineered tissues can serve as an alternative to animal experiments, reducing the need to use animals in research and the initial phases of clinical testing.

CRISPR/Cas9 represents a major asset to generate *in vivo disease models, such as* transgenic mice. Compared to traditional methods, this approach is faster, easier, and more cost effective, and protocols consisting of the injection of a plasmid construct containing the Cas9 and gRNA in mouse zygotes are available.^71^ Another approach is to replace the nucleus of an isolated oocyte with the nucleus of a gene‐edited somatic cell,^72^ thus rendering the use of embryonic stem cells obsolete. In addition to single points,^73^ large domains can also be mutated,^74^ and multiple genes can be targeted simultaneously, with an efficiency of 95% for single mutants and 80% for double mutants.^28^ More recently, in vivo genome editing in specific tissue has been demonstrated by synthetic short‐lived gRNA‐Cas9 RNP complexes.^75^ As genetics is postulated to be a main contributor to disc degeneration and DDD,^76^ in vivo models expressing Cas9 specifically in the IVD or delivery of short‐lived RNP specifically to the IVD will be useful to better understand the role of highlighted candidate genes in disease progression. Such genes include the vitamin D receptor, aggrecan, type IX collagen, asporin, MMP3, IL‐1, and IL‐6 as polymorphisms in these genes may influence IVD degeneration mechanisms.^77^ In vivo inducible systems based on dCas9 have been particularly useful in phenotypic and epigenetic studies in postnatal mammals and in cancer models, allowing the study of gene‐level changes.^78^, ^79^ Specifically, induced epigenetic remodeling enabled the amelioration of disease symptoms in mice^80^ and thereby represents an interesting approach for the IVD. As such, targeted‐induced epigenetic changes have been performed to investigate the effects of TNF‐α and IL‐1β signaling in primary IVD cells.^53^

Mice (and small animals in general) are not the most suitable models to simulate IVD pathologies and therapeutic testing. Unlike human IVDs, their IVDs are too small, contain notochordal cells with self‐regenerative potential, and fail to mimic the diffusion limitations of the human IVD. On the other hand, IVDs of larger animals (eg, sheep or dog) have comparable biology and diffusion distances to humans.^81^ CRISPR/Cas9 is not limited to mice but has also been used in domestic species, such as pigs,^72^, ^82^ sheep,^83^ goat,^84^ and cattle,^85^ as well as dogs^86^ and nonhuman primates.^87^ While structural models of IVD degeneration, including injury or chemical treatments, are invasive and unrepresentative of the natural pathophysiology, CRISPR/Cas9 may allow a spontaneous simulation of the disease, for example, by activation of senescence pathways. In the future, large animals with inducible Cas9 in their IVDs can function as programmable translational models for therapeutic testing.

Besides serving the purpose of translational medicine, animal models are also used for research in developmental biology. Notably, notochordal cells are currently of great interest in the IVD scientific community. In the field of spine and cartilage development, the zebrafish is a useful model and a powerful tool for in vivo CRISPR‐based screenings.^88^ CRISPR/Cas9 *kinesin‐I* deletion in zebrafish revealed the role of this gene in cartilage remodeling and chondrocyte maintenance during craniofacial morphogenesis,^89^ while CRISPR/Cas9 knockout of *cavin1b* in the embryonic notochord showed that caveolae, which are formed by Cavin1b, mediate the mechanoprotection of notochordal cells during development.^90^ Recently, a zebrafish model of idiopathic scoliosis was created by CRISPR/Cas9 deletion of *mapk7.* 91

### 2.2 CRISPR/Cas9 in preclinical testing for IVD therapy

For the efficient treatment of painful IVD degeneration, the pathways that mediate inflammatory and catabolic responses, pain sensing, cell resistance to oxidative stress, and production of ECM should be targeted. For example, CRISPR/Cas9 can enhance the expression of growth factors and ECM proteins, reduce the expression of inflammatory mediators, or correct unfavorable gene polymorphisms in affected individuals, providing patient‐specific treatments.^77^ To achieve this, precise reprograming of gene expression and repression is needed together with user‐friendly protocols that allow for clinical translation. Importantly, it has been demonstrated that CRISPR/Cas9 can simultaneously activate, repress, and knockout several distinct genes in a single cell.^92^ This possibility of CRISPR/Cas9 for multiplexing can be a major step forward in the treatment of multifactorial and degenerative diseases such as IVD pathologies.

Another type of Cas protein, Cas13, can be used to edit RNA by targeting CRISPR/Cas13 to RNA instead of DNA^39^, for example to correct alternative splicing. It has been shown that certain splicing variants of fibronectin mRNA may be associated with IVD degeneration.^93^ Alternative splicing of multiple mRNAs is also involved in chondrogenic differentiation in response to hypoxia, but no such data exist for IVD cells.^94^ Specific RNA editing by CRISPR may not only be used to correct false alternative splicing, but also mimic protective alleles, or guide differentiation of stem cells. CRISPR‐based genome modification of IVD can be performed directly in vivo or indirectly ex vivo in therapeutic cells that are subsequently transplanted into the IVD. Although targeted CRISPR/Cas9‐mediated transgene integration would be ideal, it is not yet completely feasible, and current delivery methods have to be improved.

#### 2.2.1 Ex vivo edited autologous IVD cells

Autologous IVD cells can be obtained from biopsies removed during surgeries. These cells are frequently affected by pre‐existing degeneration and suffer from poor expansion rates in vitro.^95^, ^96^ Nevertheless, IVD cells subjected to conventional adenoviral gene delivery of TGF‐β, BMP‐2, BMP‐7, or sex‐determining region Y box 2 (SOX2) demonstrated the ability to restore the proteoglycan content and modulate the biological processes in vitro and in vivo,^97^, ^98^ suggesting that precise gene targeting in degenerated IVD cells is also possible. Recently, human articular chondrocytes with stable CRISPR/Cas9 knockout of IL1R1 were prepared in vitro and found to have superior properties over nonedited therapeutic cells,^99^ with recent evidence that TNFR1 and IL1R1 can similarly be targeted via epigenome editing in human primary IVD cells.^53^ This suggests that deletion or knockdown of IL1R1 in therapeutic cells may improve the outcome of cell therapies for patients suffering from joint diseases.

#### 2.2.2 Ex vivo edited stem cells

**Figure 3:**
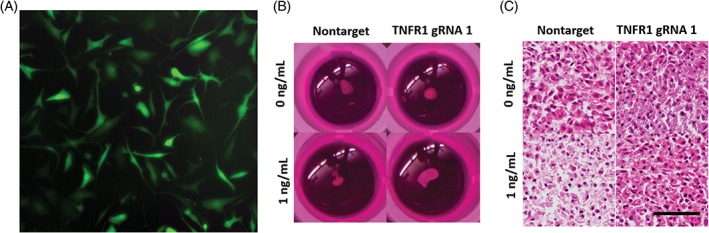
CRISPR epigenome editing in primary IVD cells and stem cells. (A) Lentiviral transduction of primary human IVD cells (69 years, female) expressing CRISPR epigneome editing system.^53^ Protection of adipose tissue‐derived stem cells from TNF‐α after CRISPR epigenome editing of TNFR1, as demonstrated by (B) pellet size and (C) H&E staining of pellets^106^

Adult stem cells, such as bone marrow mesenchymal stromal/stem cells (MSCs) or ASCs, can activate hallmarks of IVD regeneration.^100^, ^101^ Some of their advantages include high proliferation rates, and thus the possibility to expand cells with target modifications. Implantation of adult stem cells in animal models has resulted in the restoration of an IVD‐like phenotype, and promising outcomes were found in pilot clinical applications (phase I/III clinical trials).^102^ However, the major drawback of stem cell‐based therapies is the poor survival rate of implanted cells due to the pre‐existing catabolic and inflammatory environment in the IVD.^103^, ^104^, ^105^ Farhang et al. recently used *CRISPR/Cas9 epigenome editing* to modulate inflammatory responses of human ASCs by repressing the expression of cytokine receptors TNFR1 and IL1R1. Using dCas9‐KRAB‐induced, site‐specific H3K9 methylation of TNFR1 and IL1R1 promoters, resistance of ASCs to inflammatory environments was achieved, thus demonstrating the ability of CRISPR/Cas9 epigenome editing to modulate inflammatory signaling in implantable cells (Figure 3).^106^ Although this method does not produce complete knockout of gene expression, these systems have the advantage of being inducible and reversible.

In murine iPSCs subjected to chondrogenic differentiation, only complete homozygous *CRISPR/Cas9 knockout* of IL1R, but not heterozygous knockout, resulted in a cytokine‐resistant cartilage phenotype without chronic inflammation.^107^ Taking this approach further, an inflammation‐resistant cartilage with the ability to autonomously regulate its own inflammatory responses was recently engineered by *CRISPR/Cas9 gene editing.* In murine iPSCs, sequences for cytokine antagonists IL1Ra and sTNFR1 were targeted to a first exon of the proinflammatory chemokine (C‐C) ligand 2 (Ccl2), a common target of TNF‐α and IL‐1β. In response to cytokine stimulation, the edited cells produced TNF‐α and IL‐1β antagonists and thus self‐inhibited cytokine‐mediated signaling.^108^ This approach demonstrated the potential of CRISPR/Cas9 gene editing for the autonomous delivery of anti‐inflammatory agents involving cellular feedback loops.

Apart from IVD inflammation, therapeutic cells are also subjected to other microenvironmental challenges, such as low pH, nonphysiological loading, hypoxia, or starvation. A possible strategy against the consequences of such a harsh microenvironment could be preventing senescence by regulating the expression of cyclin‐dependent kinase inhibitor 2A (p16), cellular antioxidant enzymes, or DNA damage pathways.^109^ CRISPR/Cas9‐modified, microenvironment‐resistant cells can be edited to coexpress for example sequences of enhanced synthesis of the ECM.

#### 2.2.3 Targeted genome editing in vivo

CRISPR/Cas9‐based agents can be delivered directly by local application to protect endogenous cells from chronic inflammation and pain, enhancing their anabolic function and thus counteracting degeneration. As IVD cells are not ideal targets for direct in vivo genetic alterations due to their scarcity and inaccessibility, especially in the adult NP, editing of other cell types can be considered.

Activation of dorsal root ganglion (DRG) neurons and subsequent pain is known to be associated with inflammatory responses of degenerated IVD.^5^ A recent in vitro study of Stover et al. explored the potential of DRG‐directed *CRISPR/Cas9 epigenome editing* to reduce low back pain (LBP).^110^ Targeted delivery of gRNA‐dCas9‐KRAB to a promoter of A Kinase Anchor Protein 150 (AKAP150) gene in DRG neurons, stimulated with catabolic IVD‐conditioned media, abolished the nociceptive activity of the DRGs while preserving their nonpathological activity. This study demonstrated the potential of CRISPR/Cas9 epigenome editing of pain‐related genes in nociceptive neurons as a therapeutic strategy for LBP.^110^ Recently, CRISPR/Cas9 adeno‐associated virus (AAV)‐based delivery into neurons was performed in a rat model of retinal dystrophy for correction of the proto‐oncogene tyrosine‐protein kinase MER (*Mertk*) gene via homology‐independent targeted integration (HITI). Although there was only partial function rescue, the results suggest the feasibility of AAV in vivo‐targeted gene therapy in neurons, with possible implications for LBP.^111^

### 2.3 Clinical translation of CRISPR/Cas9 gene therapy

CRISPR/Cas9 is already at various stages of clinical trials for a number of disorders due to its wide applicability and ease of customization. The first application of therapeutic genome editing has entered clinical testing for editing of the C‐C motif chemokine receptor *CCR5* in CD4 T‐lymphocytes of HIV‐positive patients.^112^ Despite the difficulties, clinical trials based on adenoviral‐mediated ZFN gene delivery into T‐lymphocytes were completed. A new planned trial investigating the safety of the ex vivo *CCR5* modification in CD34+ hematopoietic stem cells by CRISPR/Cas9 is ongoing in HIV‐positive patients to prevent AIDS development (clinicaltrial.gov: NCT 03164135). Another CRISPR/Cas9 application in humans is the ex vivo‐programmed cell death protein 1 (*PD1*) gene deactivation in T‐lymphocytes of a lung cancer patient and subsequent introduction of the edited cells into the patient, which was based on previous encouraging in vitro results.^113^, ^114^ *PD‐1* gene modification is a promising approach that should be clinically trialed for other malignancies, but caution in trial designs with CRISPR/Cas9 needs to be taken when it comes to ethical requirements of scientific validity.^115^

The clinical application of CRISPR/Cas9 machinery directly in the human body is envisioned for the treatment of HPV‐related cervical intraepithelial neoplasia (clinicaltrial.gov: NCT03057912). A plasmid containing CRISPR/Cas9 is designed to target the gene for viral E6/E7T1 protein and thus eliminate the viral genes from cells infected by HPV and prevent their malignant transformation. Currently, a major challenge for using CRISPR/Cas9 in vivo is the limitation of viral delivery by vector size, possible immunogenicity, safety concerns, and regulatory hurdles. Integrase‐deficient lentiviral vectors (IDLVs) for delivery of CRISPR/Cas9 components have been recently developed.^116^ Although progress has been made, developing better delivery systems, together with development of strategies, to systematically minimize off‐target effects is necessary for the therapeutic advancement of CRISPR/Cas9 in the future.^117^

Although genome editing in the IVD is still far from clinical application, ethical and safety concerns related to the use of CRISPR/Cas9 should be considered now.^118^ Off‐target effects, which are specifically high in human cells compared to other species, may jeopardize the safety of patients by deregulating other genes with high homology to the intended target DNA sequence. This may cause severe mutations with potentially detrimental consequences, such as cell death. Ethical concerns for CRSIPR/Cas9 are mostly related to heritable genetic engineering, that is, genome editing in the human germline, and the associated possibility that introduced modifications can be transmitted through generations. While techniques for genome engineering thus far were limited to somatic cells and hence restricted to patients accepting the risk of possible side effects after informed consent, CRISPR/Cas9 may lead to unpredictable, possibly dangerous changes in later generations that the consenting patient would be responsible for. However, the extent of the risk depends on the treated cell and tissue type and is likely less pronounced for intradiscal applications.

Clinical translation of CRISPR/Cas9 for IVD pathologies holds promise as this will allow targeting of the underlying molecular processes contributing to disc degeneration and pain development and hence constitute a major improvement compared to current, symptom‐driven therapies. While approaches for CRISPR/Cas9‐based IVD regeneration may be challenging to translate to clinical practice due to unfavorable risk‐benefit ratios^119^ (especially when considering that degeneration itself does not always induce suffering or pain), treatments that specifically target disc‐related pain have higher chances for translation. Both CRISPR/Cas9‐based regulation of inflammatory processes within the IVD and modification of nociception in spinal nerves may have the highest potential for clinical application due to the high socioeconomic burden of LBP.^120^ Furthermore, the means to enhance the mechanical stability of the AF through genome editing and thus prevent IVD herniation would be of great significance for the healthcare systems,^121^ but this approach would strongly rely on (currently not existing) the early identification of patients at risk. However, when designing new CRISPR/Cas9 treatment strategies, the limitations of this technique for patients in whom cells have already undergone alterations in cell number, activity, and phenotype will have to be considered. Hence, appropriate patient selection, depending on the respective target gene, will be an important factor in the clinical translation of genome editing in the IVD.

## 3 CONCLUSION

While our understanding of the mechanisms of the CRISPR/Cas9 system and its application in the clinical setting are still developing, CRISPR/Cas9 has the potential to induce a paradigm shift in the study and treatment of human diseases, including DDD and LBP. CRISPR/Cas9 systems provide a novel tool to improve the modeling of DDD and allow studying functions from a macroscopic (body) to a microscopic (cell) scale across all mammalian species. Modeling of IVD degeneration has always been a challenge and is a limitation to the field's progression that may be overcome by CRISPR/Cas9. In addition, gene editing, knockout, and endogenous gene expression control represent powerful tools to advance cell engineering in novel and efficient ways, hindered thus far by technological limitations, complexity of applications, and/or the expense/rapidity of development. This will have profound effects on both cell therapeutics and gene therapies for IVD degeneration application. However, the field has much to learn about the delivery and safety of these systems. Despite these challenges, CRISPR/Cas9 represents a promising new tool that has the potential to break through technological barriers that have been impeding the field's progress and therefore change our thinking and treatment of IVD degeneration, DDD, and LBP.
